# “You Need to Set a Daily Schedule”: Understanding Successful Aging via Three-Sided Viewpoints

**DOI:** 10.3390/healthcare11223005

**Published:** 2023-11-20

**Authors:** Michal Tsadok-Cohen, Sara Rosenblum, Ortal Cohen Elimelech, Simona Ferrante, Sonya Meyer

**Affiliations:** 1 Department of Occupational Therapy, Faculty of Social Welfare and Health Sciences, University of Haifa, Abba Khoushy Ave 199, Haifa 3498838, Israel; rosens@univ.haifa.ac.il (S.R.); ocohe106@campus.haifa.ac.il (O.C.E.); 2 Department of Electronics, Information and Bioengineering, Politecnico di Milano, 20133 Milano, Italy; simona.ferrante@polimi.it; 3 Department of Occupational Therapy, Faculty of Health Sciences, Ariel University, Ariel 40700, Israel; sonyam@ariel.ac.il

**Keywords:** focus groups, self-management, diversity, older adults, self-tailor

## Abstract

This study aimed to identify the constructs related to successful aging in the context of engagement in social and productive activities. A qualitative design was used to explore three viewpoints on the aging period with 9 focus groups (3 each for adults 65 years or older, older adults’ family members, and health professionals) and 18 interviews with older adults (older adults M_age_ = 71.7 years, Standard Deviation = 4.62). The transcripts were analyzed using constructivist grounded theory principles. Three categories were identified for successful aging: (a) engagement with life, including social participation, fixed/flexible schedule, time, and meaningful occupation; (b) self-management abilities, including producing daily schedules, independence, and initiation/striving toward goals; and (c) diversity among older adults, including their views on retirement, being active or not, and their dreams/values/goals. Considering the diverse nature of older adults, recognizing the importance of life engagement and self-management abilities emphasizes the necessity for an occupation-based, self-tailored approach to enable successful aging.

## 1. Introduction

The world population is aging more rapidly than before [1], calling for a deeper understanding of older adults’ health conditions, quality of life, and feasibility for successful aging. Although most older people do not expect absolute health throughout their later years, many wish their health/physical condition would enable them to continue working for as long as they like, socialize, remain independent, and look after themselves [2].

Health promotion frameworks refer to social determinants of health and highlight the importance of participation and engagement. A cross-cultural comparison of older adults’ perspectives on successful aging emphasized the value of social engagement and positive attitude [3]. Individuals’ engagement in occupations they choose and that are meaningful for them leads to health, well-being, and participation in life [4]. The Model of Human Occupation points to the significance of the motivation for occupation (volition), the importance of roles and routines, and the physical and mental abilities that underlie skilled performance [5]. However, aging is accompanied by chronic health conditions and a decline in mental abilities and executive functions, which can significantly affect the participation levels of older adults [6,7]. Therefore, older adults are challenged to face changes in these components as they strive for successful aging.

There are various definitions but no universal operationalization or standardized metric of successful aging [8,9]. Despite its outdated source, Rowe and Kahn’s [10] definition seems the most common. Their definition is based on a distinction between usual and successful aging [11,12]. Rowe and Kahn [10] defined successful aging as a multidimensional term comprising three components: (a) absence of illness or illness-related disability, (b) preserved physical and cognitive capacity, and (c) engagement in social and productive activities. Research has frequently related the term to physical functioning and health. Only later definitions extended the focus to well-being, social engagement, and personal resources [8]. 

However, understanding the interaction among day-to-day experiences, health, and well-being still needs to be improved [13]. Considering Rowe and Kahn’s [10] third component—engagement in social and productive activities—most studies individually investigated specific, isolated segments of engagement, such as physical activity [14], education [15], social participation [16], or leisure [17]. Exploring this component as a whole or amplifying its meaning to a broader concept is lacking. Moreover, the literature on older adults’ time use is scarce [18,19]. How older adults spend their time and build their schedules is crucial and warrants further understanding, mainly about its association with successful aging. 

Most concepts of successful aging reflect the investigators’ lenses [20], and a profound understanding of older adults’ subjective viewpoints must be developed. One systematic review analyzed critiques of successful aging models and grouped them into four categories: (1) a group that offered adding missing criteria; (2) a group that advocated for more just and inclusive frameworks that promote diversity and avoid stigma; (3) a group that presented alternative ideal models frequently rooted in Eastern philosophies; (4) a group that focused on the Missing Voices, the subjective perception of successful aging from older adults. This focus on the Missing Voices highlights the need for additional aging criteria derived from older adults’ perspectives [21]. Moreover, it underscores the necessity for increased research involving diverse older populations and under-represented populations [22,23]. Considering the Missing Voices critiques, representing the need to listen to older adults’ perspectives, recent studies included focus groups and interviews with older adults, e.g., [24]. These studies were innovative because they listened to older adults; however, they usually contained only one viewpoint; older adults, e.g., [25] or professionals, e.g., [26].

A significant difference between self-ratings of successful aging and ratings based on objective criteria has been found. In a sample of older urban African Americans, almost two-thirds (62.7%) rated themselves as aging successfully, while only 29.9% of the participants met the objective criteria for successful aging [27]. Strawbridge et al. [28] conducted a study including 867 older adults and found that 50.3% of the participants rated themselves as aging successfully in contrast to only 18.8% who were classified as successful agers according to Rowe and Kahn’s criteria. It seems that older adults often view successful aging more optimistically than clinicians and researchers, and they tend to hold a more realistic view of this domain [29]. This tendency, along with the significance older adults attach to their sense of independence and control, implies the need for self-tailoring their individual lifestyles to fit their view of success [30,31,32].

Interestingly, individual perspectives on successful aging change over time [33], and quality of life was found to be influenced by the individual’s characteristics, relationships, and dynamics [34]. Older populations are characterized by great diversity [35,36], leading to the necessity of individually tailored care [37].

In light of Rowe and Kahn’s [10] model of successful aging, our study aimed to map the primary constructs related to aging successfully in the context of engaging in social and productive activities. The current study is unique in that it combines different viewpoints and adds a third population—family members of older adults—to help comprehensively understand the complex phenomenon of successful aging. To our knowledge, no previous studies presented three-sided viewpoints on successful aging. The goals were to identify older adults’ occupational characteristics, time use, unique perspectives, resources, needs and goals, and to highlight their diversity, thereby emphasizing the need for tailored care.

## 2. Methods

### 2.1. Design

The value of qualitative research is increasingly recognized due to its contribution to understanding and representing various unidentified perspectives [38]. Our study used a qualitative design to explore three viewpoints on aging and identify the main constructs related to successful aging. Because a single study method is unlikely to shed sufficient light on multifaceted phenomena such as successful aging [39], our study consisted of focus groups and interviews. Triangulation (multiple data sources) is helpful for deeply understanding complex constructs and reducing potential bias in the study results [40]. Therefore, our data sources were diversified among three populations (older adults, professionals, and family members). The focus groups and interviews were conducted in Israel between February 2021 to July 2022, a period characterized partially by COVID-19 lockdowns. The focus groups and interviews were carried out alternately, contributing to the theoretical sampling process by adapting the questions (as discussed later). 

### 2.2. Setting and Participants

All groups and interviews were conducted online (via Zoom), apart from one face-to-face interview. The study was part of a large study under the European Union’s Horizon 2020 (ESSENCE) research and innovation program. The University of Haifa Ethics Committee provided ethical approval (086/21). All participants signed online informed consent before participating. The data were electronically stored on a password-protected computer after the lead author de-identified the transcripts and assigned pseudonyms. In addition, all potentially identifiable information was removed from the selected quotes.

For this study, we defined older adults as 65 years or older. The focus group sample included three populations, with three groups each:

1. Independent older adults (20 total) living at home or in assisted living facilities and able to use a computer and Zoom independently. Exclusion criteria were physical or cognitive disabilities.
2. Family members of older adults (19 total) familiar with the older adult’s everyday routine.
3. Health professionals (20 total) working with older adults for at least 5 years.

Eighteen additional older adults meeting the same inclusion and exclusion criteria participated in personal in-depth interviews.

Of the 38 older-adult participants in the focus groups and interviews, M_age_ = 71.7 years, Standard Deviation (SD) = 4.62, 21 were women, and 17 were men. Almost 66% were married with children and their average years of education was 15.6 years. A significant number of them (81.5%) were retired, with an average of 8.1 years (range 1–23 years) since retirement. Nineteen family members (16 women) participated in the three focus groups. Many of the family members were children of older adults (78.9%) and intimately acquainted with their older-adult family member’s daily routines (84.2%). Among the 20 participating health professionals were two doctors, four nurses, six occupational therapists, three physiotherapists, three social workers, and two day center directors. On average, the professionals had 17.9 years of experience (SD = 9.49), ranging from 5 to 35 years, in working with older adults.

### 2.3. Data Collection

All participants were recruited via social media and by a word-of-mouth convenience sample and completed a demographic questionnaire. Nine focus groups and 18 interviews were conducted, each lasting 60 to 90 min. Two certified occupational therapists moderated the semistructured focus groups, and the first author moderated the interviews. A written guide to navigate the groups and interviews was created (Supplementary Materials: Focus group interview guides. See sample questions in Table 1) based on relevant literature to map older adults’ occupational characteristics, needs, values, and preferences. Initially, open-ended questions were asked. The questions became more focused as the transcripts were coded. No new study codes or categories emerged after nine focus groups and 18 interviews, indicating that saturation was reached.

### 2.4. Data Analysis

Constructivist grounded theory principles—in which the researcher seeks to understand a human phenomenon inductively—led the data collection and analysis. This theory consists of systematic yet flexible guidelines for collecting and analyzing qualitative data to build theories from the data [41]. The qualitative coding was guided by principles of grounded theory defined by Creamer [42] and Gutterman et al. [43]. It involved several key elements such as iterative analysis process that included constant comparison of data and codes. For instance, when a new code emerged from new data, it was compared with existing data. In addition, memos were included in the cyclical process to help construct the three primary categories. Also, theoretical sampling occurred by adapting the interview questions, such as adding questions related to the older adults’ daily routines or seeking new characteristics [44]. For example, we might seek participants who identified as “unsuccessful agers” or did not identify as religious to provide a complete and comprehensive understanding.

The focus groups and interviews were recorded and transcribed verbatim. The transcripts were then compared with the recordings to verify reliability. The coding and analysis process was undertaken in two phases. In Phase 1, all data gradually accumulated from the focus groups and interviews were named and coded according to their implementation order. In Phase 2, the data were grouped around the most frequently used codes. This phase included focused coding, in which the researchers actively chose which codes were most significant to continue with toward theoretical conceptualization. The selected codes reached at least 75% percentage of agreement. The research team discussed issues and chose the focused coding after reaching mutual agreement. The initial results were presented to colleagues and some participants, and conclusions were drawn and considered in the final steps of the theoretical conceptualization. 

The study was conducted and analyzed with reference to the consolidated criteria for reporting qualitative research COREQ, [45] and standards for reporting qualitative research recommendations [46]. Several strategies were performed to ensure the findings’ trustworthiness and credibility. For triangulation, we interviewed participants from three populations and used two study methods (focus groups and interviews) to reduce potential bias in the study results [40]. Further, a thick description of the study sample, procedure, and results were provided to help the reader follow the details [47]. For a member check, the lead author met a few interviewees online, described the principal results, and received their feedback. Finally, negative case analysis is described in the Results.

## 3. Results 

### 3.1. Key Findings

**Figure 1:**
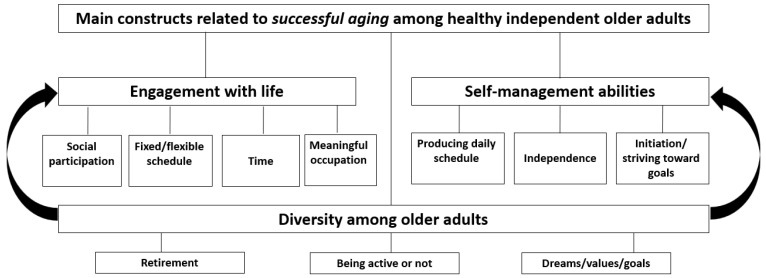
Core Categories Related to Successful Aging Among Healthy, Independent Older Adults.

Three categories were identified from both focus groups and interviews regarding successful aging among healthy, independent older adults in the context of engagement in social and productive activities: (a) engagement with life, concepts related to daily activities and participation in life, including social participation, fixed/flexible schedule, time, and meaningful occupation; (b) self-management abilities, indicating the person’s ability to direct and manage their aging process and participation in activities; this category includes producing daily schedules, independence, and initiation/striving toward goals; and (c) diversity among older adults, including their different viewpoints on concepts such as retirement, being active or not, and their dreams/values/goals (Figure 1). This third construct is also expressed by the differences among older adults in the other two constructs.

### 3.2. Engagement with Life

Participating in activities and engaging with life were among the most discussed issues in the older-adult participants. They shared their first thoughts when they woke up in the morning, and almost all described thoughts regarding the activities they planned for that day. Out of 29 older adult participants who answered directly to the question: ‘When you think of the moment you got up this morning, what were the first thoughts that ran through your head?’, 22 (76%) expressed statements including activities scheduled for the day. Examples included, “I was thinking about the tasks for today”, “I was planning to do some gardening”, “What I need to do to prepare for Passover”, and “What I should do to come to this session fresh and focused”. These quotations stress the crucial role of daily activities in older adults’ lives. As mentioned earlier, engaging in social and productive activities leads to well-being and successful aging.

#### 3.2.1. Social Participation

The three populations interviewed reflected older adults’ participation in social activities. The older adults described their social participation in detail. A newly widowed shared her engagement with her family:

“*I am not staying at home. I visit my children… Every Friday, I visit one of them; they are angry when I refuse… They do not let me [stay alone]; I come and eat with them and go out with them*”.

A granddaughter described her 89-year-old grandmother’s social participation: “The things that occupy my grandmother are mostly visiting the day center for older adults, where they can play, talk, and participate in various classes. And the fact that her family visits her always makes her satisfied”.

Similarly, a social worker described the various opportunities older adults have to participate in cultural activities: “[Older adults] enjoy activities addressed to the third age, … cinema, shows, museums, … everything related to sports, leisure, and cultural activities, … including visiting the country club and the cinematheque”.

The importance of social participation for older adults was reflected widely throughout the three populations.

#### 3.2.2. Fixed/Flexible Schedule

At the beginning of the data collection process, we were convinced that routines were fundamental to successful aging and ensuring life satisfaction. Gradually, we realized that reality is more complex. Data analysis revealed that some older adults like routines and a fixed schedule (32 of 38 older adults, 84%), whereas others (16%) prefer flexibility and changes. A 69-year-old woman shared, “Morning routine is steady. I have a routine, but it’s a very flexible routine”.

Similarly, a 73-year-old woman shared her spontaneous schedule: “There are some fixed activities. For example, on Mondays, I go out with my friends to listen to some lectures… The rest of the things are spontaneous. Like tours, movies”.

This issue also emerged in the family-member groups. One participant described her 80-year-old parents’ rigid schedule: “My parents have a pretty fixed schedule. There is a plan! It’s tough to change this plan… My dad was very flexible and creative, but now there is a plan, and you have to follow the plan”.

This category reflects the heterogeneity among older adults and emphasizes that each person has different needs and preferences. Some people need routines and a fixed schedule to feel content; others are satisfied with spontaneity. Regardless, being occupied, productive, and engaged with life was a leading factor in successful aging.

#### 3.2.3. Time

Regarding the time resource, some older adults benefitted from their spare time after retiring, whereas others had difficulty managing it. As one 69-year-old female participant noted,

“*I have a resource, and I think it’s significant after you retire—you have time! It’s a huge gift; … I will do it tomorrow if I do not do it today. Or, sometimes, I do only one or two things a day because I can do the third thing [later]… I do not need to run around myself all the time… You can do so many things with the spare time, and it’s much fun*”.

In contrast, a 74-year-old woman shared,

“*I try not to waste time, but it’s impossible to say I am always busy. I am a person who wakes up very early in the morning and starts doing; no matter what, there is always something to do… Now I have much free time; I am not occupied enough. You have days in which you have nothing but a book*”.

Similarly, a 70-year-old woman noted, “Although a year has passed (since retirement), I feel only now I start to build my desired schedule because, until now, I did things out of constraints of spare time”.

This construct of time use was also present in the family-member’ groups. One daughter shared her parents’ feelings: “They feel bored. My mom often says, ‘It’s boring here, between four walls, two old people’. This is how they feel”. On the other hand, an occupational therapist from a professionals group noted, “Some older adults manage to build a great routine. These people often complain they are too busy”. This example presents the various attitudes toward spare time after retirement. Some older adults consider it a precious resource, while others have difficulty managing it.

#### 3.2.4. Meaningful Occupation

Many older adult participants reported a meaningful occupation like paid work or volunteering. For example, a 70-year-old pensioner noted, “I retired a year ago… I have just started volunteering at Sheba Hospital twice a week, and I am very excited.” A 79-year-old man expressed the importance of being occupied in meaningful, productive activities:

“*I go to work, and I do not have to. I was supposed to retire many years ago, but I love my job and enjoy it… All my friends sit at home all day, doing nothing. But I am active! A hundred percent… I want to work! I would rather work than sit at home and think about what to do*”.

A 69-year-old daughter emphasized the meaning of volunteering to her mother: “I think the center of the week for my mom is her volunteering… Monday is ‘the holy day.‘… It’s almost the only opportunity for her to get out of the house”.

On the other hand, a 70-year-old woman described the frustration of not having the opportunity to volunteer: “Many doors were closed on me when I wanted to participate in volunteering (during the COVID-19 pandemic), and it hurt me”.

A day center director highlighted a different angle, explaining the importance of meeting new people through volunteering:

“*Independent older adults change community (after retirement). They need to find new friends in their geographic area… I think independent older adults need help there… Usually, it starts with volunteering, people looking for a good channel to get to know [people in] their immediate geographic environment.*”

The emphasis of the importance of meaningful occupations such as work or volunteering in the third age was demonstrated by the older adult participants, family members and professionals.

### 3.3. Self-Management Abilities

The importance of activities and social participation in the third age is well known. However, self-management abilities, such as producing daily schedules, independence, and striving toward goals, can help individuals carry out their plans for engagement with life and productivity.

#### 3.3.1. Producing Daily Schedules

We found diversity in the power of older adults to produce a satisfying schedule for themselves. For example, a 69-year-old former manager described his daily routine: “I write my schedule on my computer, and then my job is to fill it with activities.” Similarly, a 71-year-old woman emphasized the importance of time management:

“*I did not have any crisis when I retired… and immediately I started attending various classes… I think it’s essential to have plans for managing your day. You can do many things if you want to, and you must manage your time correctly*”.

On the other hand, a 69-year-old participant expressed difficulty producing a schedule: “Even though I retired one year ago, I failed to organize a daily schedule. So you wake up, watch some TV, walk to the beach, but it is still not a schedule that causes life satisfaction.”

The son of a 67-year-old man in a family-member group also described an example of his father’s self-management abilities:

“*He is a very busy man; he does not have any commitments, but he always creates things for himself to do… He always ensures he has something to do and does it with joy and happiness… He is a very active man… I never see him sitting and doing nothing*”.

A social worker from a professionals focus group expressed the importance of managing one’s spare time:

“*A person suddenly has so much spare time, and they do not know what to do with [it] all… They need help; how I start to motivate and manage myself while all the support systems I had from work are suddenly gone… The phone does not ring like it used to, feeling emptiness. Not everyone experiences it, of course, but this step has a potential crisis. We need to help them learn how to plan the new period, including how I motivate myself to participate in activities, get to know new friends and create new hobbies*”.

All three focus group types identified managing the aging period as essential to successful aging. One interviewee, a 68-year-old woman, painted a different picture. She gave the impression of having good self-management abilities and described her active schedule in detail, including self-care for a healthy lifestyle. Despite that, she did not seem to experience successful aging. A possible explanation could be that she was very stressed due to the COVID-19 epidemic. She shared, “I think COVID-19 added many wrinkles to my face… I actually see how I became older”. Possibly the stress and tension caused adversely affected her sense of satisfaction with life despite her good self-management abilities.

#### 3.3.2. Independence

Self-management includes an internal drive to manage oneself without depending on the environment. Older adults were asked about their independence level in various occupations. The majority reported complete independence in household (79% of participants), shopping (92%) and financial management (95%). Many of the older adult participants shared their desire to remain independent. A 73-year-old woman described,

“*I like being independent. I feel I can count on myself and feel good about not being dependent; for me, it’s imperative. Even though the people around me want to feel like they are helping me, I feel comfortable in this situation*”.

In contrast, a 69-year-old man described his friend’s unwillingness to learn new things and become independent:

“*I met a friend last week who also retired last year… I suggested he join a recommendable technological platform, but he refused. I asked him what he is afraid of, and he said, “I do not get along with these staff”. I guess this person will get stuck… This is life… We need to minimize gaps*”.

The construct of independence was not prominent in the professionals focus groups but was discussed broadly by older adults and family members.

#### 3.3.3. Initiation/Striving toward Goals

Initiative and striving toward goals are meaningful in the third age, as manifested in the older-adult and professionals groups. A 71-year-old woman stated that she had set a goal to learn to draw: “It’s a goal I set for myself, and I am working on achieving it. It’s something I really want. I do not know if I will be good at it, but at least I will give myself a chance.” Initiative can also happen in crisis periods. A 73-year-old woman said, “Even during COVID-19, we succeeded in creating a group of eight women from the area, and we used to have many walking tours.”

A day center director who is also a new pensioner shared her experience of being active in learning her environment and finding new occupations after retiring:

“*So, the self-management of getting to know your new geographic space needs support and guidance. Since I came from this field, it was not a problem for me to contact the welfare department and set an appointment to see how I could help*”.

The importance of having initiative and managing oneself was reflected in advice from a participant:

“*I have an offer for you. Do not wait for someone to contact you. You need to start looking for [employment]… You said your children live far away and do not need you, but there are many things in this world, and you do not have to watch TV all day. You need to set yourself a daily schedule*”.

This advice was given following a 69-year-old man’s description of his difficulty: “Happily, I am very independent, and I am waiting for someone to need me, to tell me to come and do this… But nobody does, and it’s a problem… You find yourself useless”.

### 3.4. Diversity among Older Adults

Older adults are not a homogenous group. Some ensure they go out for cultural activities and sports a few times a week; others prefer staying home. Some have family members nearby and spend many hours with them; others meet their family barely once a week.. In our study, the diversity among older adults was reflected in the constructs of retirement, being active, and dreams/goals.

#### 3.4.1. Retirement

Out of 38 older adult participants, 31 were retired. Years of retirement ranged between 1 to 23 years (M = 8.13, SD = 6.58). Results revealed diversity in how older adults perceive retirement. For example, a 68-year-old woman described her enjoyment of it:

“*Retirement is like heaven. I enjoy every moment; there’s a lot to do. There are many activities for the third age, and it’s worth taking part in them. The community offers many options so that you can be occupied with lectures, activities, and tours*”.

In contrast, a 69-year-old man claimed, “Nobody needs you anymore… I retired a year ago, and I still have not succeeded in organizing a daily schedule”. A 69-year-old woman presented it decisively:

“*[Working] was very good for me. Unfortunately, I was forced to retire… Since then, … I am retired, so to say, but I hate every moment… I am still trying to find my place… I feel unprepared for retirement and hate every moment without structure*”.

The challenges in retirement were also reflected in the family-member groups. A daughter of a 69-year-old man shared her father’s difficulty with the retirement process:

“*My dad is a family person but also a working person. He was in the army for many years and then worked in a big company. So the shift, the retirement, was not easy. He really had to get used to a different daily schedule; it was a big challenge*”.

A social worker from a professionals focus group also noted, “The retirement period… A person suddenly has so much spare time, and they do not know what to do with all this spare time which came without any preparation”. Interestingly, challenges with the retirement process were reflected more widely in the professionals and family-member groups than the older-adult groups.

#### 3.4.2. Being Active or Not

The diversity among older adults reflected sharply in their activity levels. A 69-year-old woman described the difference between herself and her friends:

“*I am a very passive individual. It’s funny because at work I used to be very active. However, in my personal life, I am very passive and not curious enough, I guess… We have… very good friends who are both retired and discovering life. I mean they travel all the time, and go, and see… A kind of activeness. They create a life for themselves*”.

Family members also expressed this diversity. A daughter of a 66-year-old woman shared. “She retired early from her free will and did not seem to be looking for things to do all the time… She enjoys her retirement so much”. Conversely, the daughter of a 73-year-old woman described her mother:

“*My mom is hyperactive… She wakes up at 4 in the morning, … cleans the house, … goes walking, … then goes out with her friends… Many times, she comes to visit her children and us… She is always very busy taking care of us… Shortly, she is very, very involved… She is in action all the time… She travels a lot with a group, … and it all started after she retired. Until then, she was busy at work all the time*”.

This topic also emerged from the professionals focus groups. An occupational therapist shared her experience:

“*I can say, if I am looking at independent older adults, … people manage their [lives] differently. Some older adults seek occupations and participate in classes, trips, and volunteer activities… And there are those who, after retirement, prefer to leave behind many of their occupations and are primarily engaged with householding and caring for their grandchildren*”.

A social worker from a different focus group summed up, “There are older adults who sit at home all day doing nothing, and those who go out and have an active life”.

#### 3.4.3. Dreams/Values/Goals

Hearing the different perspectives of older adults’ dreams/goals was interesting. The importance of health, independence, and family arose from many older adults:

“*The most important thing is health. With health, you can do whatever you want, whenever you want… You need to be healthy to travel and use these healthy years to do all the things we could not do because we worked hard. Family is absolutely on top priority… I did not have enough time to dedicate to my grandchildren, so now I have more time for them*” (68-year-old man).

Likewise, many professionals indicated that “staying healthy” was a main goal of older adults. However, a paramedic who treats older adults added, “They need to search for meaning, … not just wasting time and relieving loneliness and boredom. They seek something valuable so they have a reason to wake up in the morning. They need to be relevant”.

The family members showed more diversity in what they considered essential for their parents or grandparents. Examples included flying to Europe, acquiring an education, working, and retaining their cognitive capacity. In addition, they described more common goals. The daughter of a 74-year-old woman said, “The most important thing for my mom is the family, … to feel she is still relevant and a part of this circle, … that she is engaged in our lives”.

Similarly, an 89-year-old woman’s granddaughter shared:

“*My grandmother wants everyone [from the family] to be with her. Since I know her, I know she needs to have her family around her… And her grandchildren actually visit her every day… This is what makes her satisfied, the most important thing for her*”.

Finally, an 83-year-old man’s daughter referred to health as the key to other goals: “I think… they really want to stay healthy. Because for them, it’s the main key to independence… All they want is to be independent and able to travel… So their ambition is to stay mobile… and see the world”.

## 4. Discussion

Successful aging has been studied extensively, but most concepts reflect the investigators’ viewpoints [20]. To our knowledge, this is the first qualitative research exploring successful aging through a three-sided viewpoint. In our study, positive energies generally emerged from the older-adult focus groups, and more negative ideas about the aging period emerged from the family-member and professionals’ groups. This finding might be related to the notion that older adults tend to have a more realistic view of successful aging that highlights the experience of difficulties using positive emotions, whereas professionals’ perspectives focus on the avoidance of vulnerability [29]. The older adults described their daily activities in detail and most of them stated that their first thoughts in the morning involved activities planned for the day. They emphasized the activities’ significance and contribution to their satisfaction with life. However, the professionals and family members tended to focus on the difficulties and challenges of the aging period.

Engaging in meaningful occupations is essential for health, well-being, and participation in life [4]. The motivation for a chosen occupation, alongside the roles and routines in everyday life, are significant for every individual [5]. The subject of participation and daily activities under the engagement with life category was unsurprising, considering the power of meaningful occupations and the importance of being engaged with life. This category is also known as one of the core components of successful aging [10].

The three viewpoints introduced the category of self-management, which was innovative and surprising. The questions in the interview guide did not refer to concepts such as executive functions or self-management. However, the data analysis highlighted this significant component of successful aging. The cognitive domains required for self-management, such as planning, initiation, and problem-solving (i.e., executive functions), are often considered challenging capacities for older adults [7]. Executive function components significantly contribute to the participation levels of older adults [6], and the critical construct of self-management serves as a basis for the engagement with life construct. The third construct, diversity among older adults, leads us to the need to tailor successful aging for each individual.

Individually tailored care aims to promote patients’ care experiences and health outcomes by considering their individual needs and preferences in an intervention plan [37]. Because the results showed a wide diversity in daily activity patterns, tailoring a unique suit for each individual to fit their needs and preferences is warranted. However, adding the self-management category and its importance to successful aging leads to the understanding that individually tailored care is insufficient. Self-tailored care is needed.

Unlike traditional tailoring, self-tailoring is performed by the person based on the principles of self-management skills and knowledge and appropriately applying them to oneself [31]. The term self-tailoring was proposed for interventions offered to a heterogeneous group of users with no professional tailoring the intervention to separate users. In these cases, each user tailors the intervention and chooses the content that suits their situation and needs [32]. This study’s results show the significance for many older adults to be independent and in control. Previous research has also showed that self-tailoring is essential to enhance a sense of ownership and control in managing complex situations and may lead to improved outcomes [30,31]. Considering the diverse nature of older adults, recognizing the importance of life engagement and self-management abilities emphasizes the necessity for an occupation-based, self-tailored approach to enable successful aging.

## 5. Limitations

Alongside this study’s important understanding and contributions to the existing literature, it has limitations. First, the participants were primarily interviewed at distance via Zoom software version 5.5.0 (12454.0131), thus influencing the interpersonal relationship between the researchers and the participants. Moreover, this platform was apparently directed mainly to independent older adults who successfully managed technology changes. Second, the data collection occurred during the COVID-19 pandemic, suggesting impacts on participants attitudes and insights. Nevertheless, the pandemic can be viewed as an example of a stressful reality and different ways of handling it. Third, the sample was not randomly selected. Lastly, given the qualitative nature of the study, the findings cannot be generalized to other contexts.

## 6. Conclusions

Because the world population is aging rapidly, a deeper understanding of achieving successful aging is crucial. Built on existing successful-aging models, this study’s findings underscore the importance of broadening these models, highlighting older adults’ perspectives, and including self-management components to support older adults in planning their new stage of life.

Older adults who succeed in managing their daily lives and experience control of their own life delineate successful aging. The diversity among older adults supports the premise that each one needs an accurately different proportion of the constructs presented, corresponding to their needs, values, and preferences, to feel satisfied with their aging. Each needs to tailor their self-fitted “suit” to wear as they stride successfully into their older years.

In summary, Engagement with life and self-management abilities are both important factors for successful aging. The diversity among older adults leads to the need for a self-tailored approach. Future research will focus on constructing guidelines for self-tailored successful aging.

## Figures and Tables

**Table 1:** Sample questions from the moderator guide.

Individual interviews When you think about your day, do you have a set routine? Could you please describe a typical day, from getting up in the morning until you go to bed? When you think about things in everyday life that you find difficult to do, what helps you do them?
Older adult focus groups When you think of the moment you got up this morning, what were the first thoughts that ran through your head? When you think about your daily routine, from getting up in the morning until you go to bed, when do you need help? In what situations?
Family member focus groups When you think in general about the life goals of your family member, do you know where they are aiming? Where would they like to be in a year? In five years? Does your family member use technology in their daily routine?
Professional focus groups When you think about the older adults you work with, what are the types of activities they are offered or participate in? What is the thing that most disturbs or worries the older adults you work with in their daily life?

## Data Availability

The data presented in this study are available on request from the corresponding author. The data are not publicly available due to ethical restrictions.

## Supplementary Materials

The focus group interview guides can be downloaded at: https://www.mdpi.com/article/10.3390/healthcare11223005/s1.
