# Supplementary material for: “You Need to Set a Daily Schedule”: Understanding Successful Aging via Three-Sided Viewpoints
Source: Healthcare (Basel). 2023 Nov 20;11(22):3005. doi: 10.3390/healthcare11223005 (PMC10671344; doi:10.3390/healthcare11223005)
Supplement: Supplementary file 1 [file healthcare-11-03005-s001.zip › healthcare-2695239-supplementary.pdf]

Interview guide for older adults (individual interviews)

<u>Opening question:</u>

When you think of the moment you got up this morning, what were the first thoughts that ran through your head?

<u>Key Question #1:</u>

When you think about your day, do you have a set routine? Could you please describe a typical day for you, from getting up in the morning until you go to bed? What actions make you happy? Do you feel insecure when performing certain actions? Would you like assistance with different situations/actions you perform?

<u>Key Question #2:</u>

When you think about the time you invest in the things you **want** to do versus the time you invest in the things you **need** to do, is there a difference between them in terms of the energy required? The degree of fatigue? Things where time crawls or passes quickly?

<u>Key Question #3:</u>

When you think of things in everyday life that you find difficult to do, what helps you do them? How do you perform activities such as money management, shopping? travelung? Searching for information? How do you tend to react when you fail to do what you expect of yourself or what the environment expects of you? Does it happen often or rarely?

<u>Transition question:</u>

When you hear the term "Covid period", what do you think of? What thoughts come to your mind?

<u>Key Question #4:</u>

How do you feel that the Covid affected your daily routine compared to the routine before Covid?

<u>Key Question #5:</u>

When you think about your life, what is important to you in life today? How is your relationship with family members today? with friends? What do you feel you are missing today?

<u>Transition question:</u>

Do you use technology in your daily routine? What devices do you use (mobile phone, tablet, laptop, desktop)? What use do you make of them? How often do you use these devices? Do you have difficulty using technologies? What is the difficulty?

<u>Key Question #6:</u>

During the Covid period, especially during the lockdown, were there changes in the use of technology compared to the use before the Covid period? Is it in the amount of use? Type of use? The way you used it? Has technology improved your involvement in daily activities? In what areas? Were there situations in which you felt powerless in front of technology? When?

<u>Key Question #7:</u>

What technology was missing? What could help you be able to make more efficient use of technologies? What would you like to improve? What would you like to use or do with technology but can't? Why?

<u>Final question:</u>

Out of all the topics that came up in today's discussion, what is the thing that most disturbs or worries you in your daily life?

Focus group interview guide for older adults

Opening question:

When you think of the moment you got up this morning, what were the first thoughts that ran through your head?

Key Question #1:

When you think about your daily routine, from getting up in the morning until you go to bed, when do you need help? In what situations?

Key Question #2:

When you think about the time you invest in the things you **want** to do versus the time you invest in the things you **need** to do, is there a difference between them in terms of the energy required? The degree of fatigue? Things where time crawls or passes quickly?

Key Question #3:

When you think of things in everyday life that you find difficult to do, what helps you do them? How do you tend to react when you fail to do what you expect of yourself or what the environment expects of you? Does it happen often or rarely?

Transition question:

When you hear the term "Covid period", what do you think of? What thoughts come to your mind?

Key Question #4:

How do you feel that the Covid affected your daily routine compared to the routine before Covid?

Key Question #5:

When you think about the beginning of the day in the Covid period and before the Covid period, did you feel that there was a reason to get up in the morning? What is the reason?

Transition question:

Do you use technology in your daily routine? What devices do you use (mobile phone, tablet, laptop, desktop)? What use do you make of them? How often do you use these devices? Do you have difficulty using technologies? What is the difficulty?

Key Question #6:

During the Covid period, especially during the lockdown, were there changes in the use of technology compared to the use before the Covid period? Is it in the amount of use? Type of use? The way you used it? Has technology improved your involvement in daily activities? In what areas? Were there situations in which you felt powerless in front of technology? When?

Key Question #7:

What technology was missing? What could help you be able to make more efficient use of technologies? What would you like to improve? What would you like to use or do with technology but can't? Why?

Final question:

Out of all the topics that came up in today's discussion, what is the thing that most disturbs or worries you in your daily life?

Focus group interview guide for family members

Opening question:

When you think in general about the life goals of your family member, do you know where they are aiming? Where would they like to be in a year? In five years?

Key Question #1:

When you think about your family member's daily routine, from getting up in the morning until they go to bed, when do they need help? In what situations?

Key Question #2:

When you think about the time your family member invests in the things they **want** to do versus the time they invest in the things they **need** to do, is there a difference between them in terms of the energy required? The degree of fatigue? Things where time crawls or passes quickly?

Key Question #3:

When you think of things in everyday life that your family member finds difficult to do, what helps them do those things? How do they tend to react when they fail to do what they expect of themselves or what the environment expects of them? Does it happen often or rarely?

Transition question:

When you hear the term "Covid period", what do you think of in the context of your family member? What thoughts come to your mind?

Key Question #4:

How do you feel that the Covid affected your family member's daily routine compared to the routine before Covid?

Key Question #5:

When you think about your family member, what is important to them in life today? How is their relationship with family members today? with friends? What do they feel they are missing today?

Transition question:

Does your family member use technology in their daily routine? What devices do they use (mobile phone, tablet, laptop, desktop)? What use do they make of them? How often do they use these devices? Do they have difficulty using technologies? What is the difficulty?

Key Question #6:

During the Covid period, especially during the lockdown, were there changes in the use of technology by your family member compared to the use before the Covid period? Is it in the amount of use? Type of use? The way they used it? Has technology improved their involvement in daily activities? In what areas? Were there situations in which they felt powerless in front of technology? When?

Key Question #7:

What technology was missing for them? What could help them be able to make more efficient use of technologies? What would they like to improve? What would they like to use or do with technology but can't? Why?

Final question:

Out of all the topics that came up in today's discussion, what is the thing that most disturbs or worries your family member in their daily life?

Focus group interview guide for professionals

Opening question:

When you think about the older adults you work with, what are the types of activities they are offered or participate in?

Key Question #1:

When you think about the older adults' daily routine, from getting up in the morning until they go to bed, when do they need help? In what situations?

Key Question #2:

When you think about the time older adults invest in the things they **want** to do versus the time they invest in the things they **need** to do, is there a difference between them in terms of the energy required? The degree of fatigue? Things where time crawls or passes quickly?

Key Question #3:

When you think of things in everyday life that the older adults you work with find difficult to do, what helps them do those things? How do they tend to react when they fail to do what they expect of themselves or what the environment expects of them? Does it happen often or rarely?

Transition question:

When you hear the term "Covid period", what do you think of in the context of the older adults you work with? What thoughts come to your mind?

Key Question #4:

How do you feel that the Covid affected the older adults' daily routine compared to the routine before Covid?

Key Question #5:

When you think about the older adults you work with, what is important to them in life today? How is their relationship with family members today? with friends? What do they feel they are missing today?

Transition question:

Do the older adults you work with use technology in their daily routine? What devices do they use (mobile phone, tablet, laptop, desktop)? What use do they make of them? How often do they use these devices? Do they have difficulty using technologies? What is the difficulty?

Key Question #6:

During the Covid period, especially during the lockdown, were there changes in the use of technology by the older adults compared to the use before the Covid period? Is it in the amount of use? Type of use? The way they used it? Has technology improved their involvement in daily activities? In what areas? Were there situations in which they felt powerless in front of technology? When?

Key Question #7:

What technology was missing for them? What could help them be able to make more efficient use of technologies? What would they like to improve? What would they like to use or do with technology but can't? Why?

Final question:

Out of all the topics that came up in today's discussion, what is the thing that most disturbs or worries the older adults you work with in their daily life?
